# Anticholinergic burden and behavioral and psychological symptoms in older patients with cognitive impairment

**DOI:** 10.3389/fmed.2025.1505007

**Published:** 2025-02-12

**Authors:** Sabrina Pistorio, Gianluca Scotto di Tella, Vittoria Canzanella, Raffaella Merenda, Gianluigi Cuomo, Paola Iacotucci, Vincenzo Carnovale, Leonardo Bencivenga, Maria Vargas, Annalaura Manes, Mauro Cataldi, Giuseppe Rengo, Michela Zanetti, Grazia Daniela Femminella

**Affiliations:** ^1^Department of Medicine, Surgery and Health Sciences, University of Trieste, Trieste, Italy; ^2^Department of Translational Medical Sciences, University of Naples Federico II, Naples, Italy; ^3^Department of Neuroscience, Reproductive Sciences and Dentistry, University of Naples Federico II, Naples, Italy; ^4^Istituti Clinici Scientifici Maugeri IRCCS-Scientific Intitute of Telese Terme, Telese Terme, Benevento, Italy; ^5^Department of Brain Sciences, Imperial College London, London, United Kingdom

**Keywords:** anticholinergic burden, behavioral and psychological symptoms in dementia, cognitive impairment, older patients, antipsychotic drugs

## Abstract

**Background:**

Drugs with anticholinergic properties are frequently prescribed to patients with cognitive impairment. The cholinergic system plays an important role in learning processes, memory, and emotions regulation. The aim of this research is to report use of anticholinergic drugs in a clinical population and to investigate the correlation between the use of anticholinergic drugs and the risk of presenting with more severe behavioral and psychological symptoms (BPSD).

**Method:**

Patients with a diagnosis of subjective cognitive impairment, mild cognitive impairment (MCI) or dementia were recruited. Screening tests for cognitive impairment (MMSE) and functional status (ADL, IADL) were performed. BPSD were evaluated with the Neuropsychiatric Inventory (NPI). The anticholinergic burden was calculated using the ACB calculator. We compared patients at low risk of anticholinergic adverse effects (ACB < 3) versus patients at high risk (ACB ≥ 3). Chi-square test and Mann–Whitney test were used to compare the two groups. A multiple linear regression was performed to identify factors associated with higher NPI score and a logistic regression model was built to identify drug classes associated with ACB ≥ 3.

**Result:**

A total of 173 patients (mean age 74 ± 7, 74 men) were included in the study; 132 patients with ACB < 3 (low risk) versus 41 patients with ACB ≥3 (high risk) were compared. No statistically significant differences were found between the two groups in terms of demographics (age, sex) and anamnestic variables (education, marital status, family history of dementia, hypertension, diabetes, smoking, dyslipidemia, atrial fibrillation, coronary heart disease and use of alcohol). Significantly higher NPI scores were found in patients with ACB ≥ 3 (mean scores 47.3 ± 34.8 versus 25.5 ± 24.6, *p* < 0.001). Patients with ACB ≥ 3 showed lower MMSE (18.5 ± 8.6 versus 22.4 ± 7, *p* = 0.004) and more IADLs lost. In the multivariate regression analysis, after adjusting for age, sex, polypharmacy and IADLs lost, only the MMSE and the ACB scores were independent predictors of the NPI score. Being on antipsychotics, antidepressants and antidiabetic drugs was associated with increased risk of higher anticholinergic burden.

**Conclusion:**

In conclusion, the anticholinergic burden might play a significant role as a risk factor for developing more severe BPSD in patients with cognitive decline, independently from their degree of cognitive impairment.

## 1 Introduction

Behavioral symptoms (agitation, aberrant motor behavior, anxiety, elation, irritability, depression, apathy, disinhibition, delusions, hallucinations, and sleep or appetite changes) (1), often referred to as Behavioral and psychological symptoms in dementia (BPSD), are prevalent in individuals with dementia, significantly impacting the well-being of both patients and their caregivers. Almost all patients with dementia will experience one or more of these symptoms during the disease course. BPSD have been associated with greater functional decline and an increased institutionalization rate. Additionally, they contribute substantially to caregiver stress and depression, along with financial problems (2). The mechanism responsible for BPSD is still unclear and the etiopathogenesis of this condition is probably multifactorial with biological (brain changes, comorbidities, and medications), psychological (personal life history, personality) and social factors (support network, living arrangements) all having a role (1). Neurochemical, pharmacological and neuroimaging evidence suggests that BPSD pathogenesis involves not only an increase in dopaminergic but also a decrease in cholinergic muscarinic central neurotransmissions (3, 4). Nonetheless the pharmacological treatment of BPSD is currently based on typical and atypical antipsychotic, as well as antidepressant drugs (5) that target dopaminergic and serotoninergic neurotransmissions but in most cases also block central muscarinic receptors, and, therefore, could be potentially detrimental for BPSD worsening the underlying dysfunction of muscarinic neurotransmission.

Moreover, many of the drugs taken by older adults with dementia for their comorbidities have intrinsic anticholinergic (aACh) properties (6, 7). Among these drugs, some are well known for their aACh properties (e.g., oxybutynin), while others have an unexpected aACh activity that is not intended for therapeutic effect (e.g., furosemide) and physicians may not always be aware of the aACh effect of the medications on the sometimes-long list of a patient.

Drugs with antimuscarinic properties used in combination may have additive effects contributing to determine the so called anticholinergic burden (ACB), i.e., the cumulative inhibitory effects on muscarinic neurotransmission. While aACh medications prescribed for their anticholinergic effects are known to carry potential aACh side effects, high cumulative ACB may also result from the concurrent use of multiple medications that individually have weaker anticholinergic effects, with duration of use and dose that should also be taken into account. Major differences do exist in the efficacy of different drugs in blocking muscarinic receptors and, therefore, several scales have been developed to measure the ACB score, incorporating factors such as serum anticholinergic activity, muscarinic receptor affinity, clinical side effects, expert opinion, and comprehensive literature review (7–10).

The detrimental effects of high ACB scores (≥3) on quality of life, morbidity and mortality in older adults are well established since they are associated with signs and symptoms of peripheral and central muscarinic blockade including constipation, vision disturbances, the reversible drop in awareness (zombie-effect), increased risk of falls and reduced life expectancy. However, only few studies have investigated whether a high ACB score correlates with BPSD occurrence and severity (11). In particular, there is a lack of evidence in real-world memory clinic populations with a specific focus on behavioral disturbances. In the present study we report the prevalence of high ACB score values in demented patients with or without BPSD among those attending our memory clinics from July 2021 to August 2023, to investigate the correlation between the use of aACh drugs and the risk of presenting with more severe BPSD. This investigation aims to explore the contribution of aACh burden on cognition, behavior, and functional abilities in individuals with cognitive impairment to inform clinicians on the possible need to mitigate aACh burden in these patients.

## 2 Methods

### 2.1 Study design and participants

A cross-sectional study was conducted on a cohort of outpatients visiting the memory clinic at the Geriatric Unit of the Federico II University Hospital in Naples, Italy, from July 2021 to August 2023. The study group included a consecutive sample of patients presenting cognitive concerns, either self-reported or reported by a family member. Patients enrolled in this study had a diagnosis of either subjective cognitive decline, as per the Subjective Cognitive Decline Initiative (SCD-I) Working Group criteria (12), mild cognitive impairment (MCI) or minor neurocognitive disorder, or dementia or major neurocognitive disorder, as per Diagnostic and Statistical Manual of Mental Disorders (DSM) 5 definition (13). All included subjects were 65 years or older, had the capacity to provide informed consent and have been on stable medications for the previous month. Patients with concomitant psychiatric disorders (i.e., schizophrenia, bipolar disorder) were excluded.

The comorbidities and risk factor (dyslipidemia, smoke, alcohol use, hypertension, diabetes mellitus, atrial fibrillation, coronary artery disease, hearing impairment, family history of cognitive disorders) were collected, as well as sociodemographic characteristics, such as age, sex, marital status and educational level. Medications taken by the patient at the time of the visit to the memory clinic were collected and aACh burden was calculated using the ACB calculator^1^, an online platform based on the German Anticholinergic Burden score (GABS) (10) and the Anticholinergic Cognitive Burden (ACB) scale (7). The website authors used a combination of these 2 scales when creating the ACB calculator. When discrepancies arose, they opted to include the higher value in the interest of safety. The ACB score for a patient is the sum of the ACB scores for all the medications they are on with potential aACh properties.

Mini Mental State Examination (MMSE) was used to assess cognitive performance. A subgroup of 112 patients was also assessed with the Addenbrooke’s Cognitive Examination-Revised (14). Presence and severity of neuropsychiatric symptoms were evaluated with the Neuropsychiatric Inventory (NPI) (15). A higher overall NPI score (maximum 144) indicates more severe behavioral disorders. Based on previous reports, a NPI score ≥ 4 indicated clinically relevant BPSD (16).

Patient functional status was assessed with the Katz Index of Independence in Activities of Daily Living (ADL) (17)and Lawton-Brody Instrumental Activities of Daily Living (IADL) (18). ADL include six activities: bathing, dressing, toileting, transferring, continence and feeding. IADL include eight activities: using telephone, shopping, meal preparation, housekeeping, laundry, use of transportation, self-administration of drugs, and handling finances.

All participants had the capacity to give their written consent to participate in the study, conducted in accordance with the Ethical standards of Helsinki Declaration. The research protocol was reviewed and approved by the Local Ethics Committee, the “Comitato Etico Campania 3” with protocol number 8/21.

### 2.2 Sample size estimation

Based on previous reports (19) on the prevalence of specific BPSD (agitation) in patients on drugs with anticholinergic properties, we calculated that to observe a difference in prevalence of BPSD of at least 22% between subjects on high vs. low ACB score, a sample size of at least 152 subjects would be sufficient, with a power of 80% and a two-tailed significance of 5%. Considering a drop-out rate of 10%, a minimum of 167 subjects should be enrolled in the study.

### 2.3 Statistical analysis

Patients were subdivided into two groups, according to ACB score (ACB < 3 low risk vs. ACB ≥ 3 high risk). The study population was described using mean ± standard deviation (SD), median or proportions, as appropriate. The Kolmogorov–Smirnov statistic was used to assess normal distribution of data. Differences in means between groups were tested by Student’s *t*-test for independent sample when variables had a normal distribution and by the Mann–Whitney *U*-test when variables had a non-normal distribution. Differences in percentages were assessed by the chi-square test. Square root transformation of the NPI scores was performed to normalize the data distribution. Multiple linear regression and multivariate logistic regression were performed to test associations between our variables of interest. For the multiple linear regression and logistic regression, the functional form of the association between continuous factors and outcome was checked and modeled using a multivariable fractional polynomial (MFP) algorithm, as previously described (20). The relative weight of each significant factor in the final model was estimated by measuring the partial contribution to the global goodness-of-fit, as measured by the global *R*^2^ for the multiple regression model and by the McFadden’s global pseudo *R*^2^ for the logistic model. Their partition over the significant predictors was obtained by the Shapley–Owen decomposition algorithm (21). A *p*-value less than 0.05 was considered statistically significant. All analyses were performed with STATA 17 (StataCorp LLC).

## 3 Results

A total of 173 patients were included in the study (Table 1). The mean age was 74 ± 7 years. Ninety-nine patients (57%) were females. The patients had a mean of 9.5 ± 5 years of education. About half had a family history of neurocognitive disorders and dementia. Most patients were married (66%). The most frequent comorbidities were high blood pressure (65%) and dyslipidemia (57%). Patients were taking on average 6.4 (±3.4) different drugs. Thirty-two subjects were diagnosed with subjective cognitive decline, 62 with MCI and 79 with dementia. One hundred forty subjects (81%) had clinically relevant BPSD, based on NPI score ≥4.

**Table 1:** Characteristics of the overall population, and divided by ACB score.

	Total	ACB score < 3	ACB score ≥ 3	*p*-value
Number of patients	173	132 (76%)	41 (24%)	/
Age (years)	74.8 ± 7.5	75 ± 7.3	74 ± 7.8	0.385
Male sex	74 (43%)	57 (43%)	17 (42%)	0.846
Years of education	9.5 ± 5	9.7 ± 5	9.1 ± 4.9	0.612
Marital status (married)	109 (66%)	84 (67%)	25 (62%)	0.585
Family history of cognitive problems	88 (51%)	67 (51%)	21 (51%)	0.959
Hypertension	113 (65%)	86 (65%)	27 (66%)	0.934
Diabetes	34 (20%)	24 (18%)	10 (24%)	0.382
Smoke	27 (16%)	19 (14%)	8 (20%)	0.430
Dyslipidemia	98 (57%)	73 (55%)	25 (61%)	0.522
Atrial fibrillation	14 (8%)	13 (10%)	1 (2%)	0.129
Coronary artery disease	21 (12%)	15 (11%)	6 (15%)	0.575
Alcohol use	16 (9%)	12 (9%)	4 (10%)	0.862
Hearing impairment	45 (26%)	37 (28%)	8 (20%)	0.267
Number of drugs	6.4 ± 3.4	5.8 ± 2.8	8.4 ± 4.2	<0.001*
ACB score	1.4 ± 1.7	0.6 ± 0.7	4.2 ± 1.4	<0.001*
MMSE	21.5 ± 7.6	22.4 ± 7	18.5 ± 8.6	0.004
ACE-R tot^#^	69.2 ± 15.9	70.6 ± 15.5	63.6 ± 16.9	0.064
ACE Attention^#^	14.9 ± 3.4	15.1 ± 3.3	14.3 ± 3.6	0.355
ACE Memory^#^	13.2 ± 6.3	13.6 ± 6.1	11.5 ± 7	0.170
ACE Fluency^#^	6.4 ± 2.9	6.7 ± 3	5 ± 2.7	0.017*
ACE Language^#^	22.8 ± 3.6	23.1 ± 3.5	21.5 ± 3.7	0.058
ACE Visual^#^	11.9 ± 3.3	12 ± 3.3	11.2 ± 2.9	0.317
Lost ADLs	1.8 ± 2	1.6 ± 2.1	2.1 ± 1.9	0.203
Lost IADLs	4.4 ± 3	4.1 ± 3	5.8 ± 2.3	0.002*
NPI	29.2 ± 29.1	25.5 ± 24.6	47.3 ± 34.8	<0.001*
Clinically relevant BPSD	140 (81%)	104 (79%)	36 (88%)	0.199

*p < 0.05; ^#^ scores available on *n* = 112 subjects.

ACB, anticholinergic burden; ACE, Addenbrooke’s Cognitive Examination; ADL, activities of daily living; IADL, instrumental activities of daily living; MMSE, Mini-Mental State Examination; NPI, Neuropsychiatric Inventory.

The aACh drugs used by the patients recruited are listed in Table 2. The most used drugs with anticholinergic burden were quetiapine, metformin, trazodone and furosemide.

**Table 2:** Anticholinergic drugs used in the study population.

Drugs	*N*. patients (%)	ACB score
Antipsychotics	36 (21%)	
Haloperidol	6	2
Quetiapine	23	3
Promazine	3	3
Perphenazine	3	3
Olanzapine	1	3
Antidepressants	45 (26%)	
Trazodone (also used as sleeping pill)	15	1
Escitalopram	13	1
Sertraline	10	1
Fluvoxamine	1	1
Mirtazapine	1	1
Amitriptyline	4	3
Clomipramine	1	3
Antidiabetics	19 (11%)	
Metformin	19	1
Diuretics	14 (8%)	
Furosemide	11	1
Chlortalidone	3	1
Benzodiazepines	13 (7%)	
Alprazolam	4	1
Lorazepam	3	1
Clonazepam	3	1
Diazepam	2	1
Triazolam	1	1
Cardiovascular drugs	7 (4%)	
Metoprolol	1	1
Diltiazem	1	1
Digoxin	1	1
Nifedipine	1	1
Atenolol	3	1
Proton pump inhibitors	5 (3%)	
Lansoprazole	5	1
Parkinson’s drugs	5 (3%)	1
Levodopa	4	1
Selegiline	1	1
Antiepileptics	6 (3%)	
Oxcarbazepine	2	2
Valproic acid	3	1
Phenobarbital	1	1
Bronchodilators	7 (4%)	
Tiotropium	4	1
Glycopyrronium	3	1
Anti-rheumatic drugs	4 (2%)	
Methotrexate	3	1
Ciclosporin	1	1
Myorelaxants	2 (1%)	
Cyclobenzaprine	2	2
Anti-inflammatory drugs	1	
Etoricoxib	1	1

ACB, anticholinergic burden.

One hundred and five patients (61%) were taking at least one aACh drug. According to ACB score, we found 132 patients at low risk of anticholinergic effects (ACB < 3) and 41 patients at high risk (ACB ≥3).

No differences were found between the two groups in terms of demographic characteristics (age, sex, years of education, and marital status), family history of cognitive problems, major cardiovascular risk factors (diabetes, smoke, dyslipidemia, atrial fibrillation, coronary artery disease), alcohol use and hearing impairment. The total number of drugs was higher in the ACB ≥3 group (Table 1).

Patients at high risk (ACB ≥3) were more likely to have lower cognitive performances (mean MMSE score 18.5 ± 8.6 versus 22.4 ± 7, *p* = 0.004), to be more functionally impaired in IADL (mean IADLs lost 5.8 ± 2.3 versus 4.1 ± 3, *p* = 0.0052), and to suffer from more severe neuropsychiatric symptoms (mean NPI scores 47.3 ± 34.8 versus 25.5 ± 24.6, *p* < 0.001). In the group of subjects who performed the ACE-R cognitive test, participants with ACB ≥ 3 had lower verbal fluency scores compared to subjects with ACB < 3 (mean Fluency score 5 ± 2.7 versus 6.7 ± 3, *p* = 0.017).

To test the hypothesis that higher ACB score might be associated with worse BPSD in this population, we performed a multiple linear regression test, with MMSE, IADL and ACB scores as predictors of higher NPI scores, and adjusting for age, sex and number of drugs (Table 3). Our model proved significant (*R*^2^ = 35%, *p* < 0.01), with MMSE and ACB scores as the only independent predictors of NPI scores in this population. 77% of the variance in NPI scores was explained by MMSE scores, while 23% of it was explained by the ACB score.

**Table 3:** Multivariate regression model for NPI scores in the study population.

	*B*	R^2^c (%)	95%CI	Functional form
Sex	−0.127		−0.919, 0.664	
Age	0.009		−0.048,0.066	
MMSE	−189*	77%	−0.251, −0.128	Non-Lin
ACB score	0.338*	23%	0.089,0.588	Lin
Lost IADLs	0.050		−0.101,0.200	
Number of drugs	0.011		−120,0.142	

*R*^2^ = 0.35; *p < 0.05.

*R*^2^*c*, global *R*^2^ contribution; Non-Lin, non-linear; Lin, linear.

ACB, anticholinergic burden; IADL, instrumental activities of daily living; MMSE, Mini-Mental State Examination.

We therefore sought to evaluate which drug classes would most likely influence the risk of being in the high risk ACB ≥ 3 group, potentially contributing to worse BPSD. At this aim, we built a multivariable fractional polynomial logistic regression model for ACB ≥ 3 vs. ACB < 3 groups (Table 4). The predictors were the following drug classes: antidepressants, antipsychotics, antidiabetics, diuretics and benzodiazepines. We included drug classes that at least 5% of the study population was on. The model proved significant with a pR2 = 0.65 (*p* < 0.01), with antipsychotics, antidepressants and antidiabetics being significant predictors. Of note, as indicated by the percentage of contribution to the global pR2, the greatest fraction is attributable to antipsychotics (85.6%), while antidepressants (8.7%) and antidiabetics (5.7%) have a more modest contribution, suggesting that not only psychoactive drugs might contribute to higher ACB scores in this memory clinic population.

**Table 4:** Logistic regression model of different drug classes versus ACB group in the study population.

	*B*	Odds = Exp (B)	pR^2^c (%)	95%CI
Antipsychotics	6.04*	420.2	85.6	71.317, 2476.466
Antidepressants	2.10*	8.2	8.7	1.830, 36.337
Antidiabetics	2.82*	16.7	5.7	3.321 83.933
Diuretics	1.25	3.5		0.370, 33.813
Benzodiazepines	1.13	3.1		0.112, 84.706

pR^2^ = 0.65 (Cox and Snell); Model *χ*^2^(2) = 122.4, *p* < 0.001; *p < 0.05.

pR^2^c, global pseudo *R*^2^ contribution.

## 4 Discussion

The main finding of the present study is that more than a half (61%) of the patients attending an outpatient visit for cognitive impairment at our clinic were using at least one anticholinergic medication and 24% of them had an ACB score of 3 or higher (7, 10).

Previous studies already reported a high prevalence of use of anticholinergic drugs in older adults on polypharmacy.

For instance, in an Italian study approximately 40% of community-dwelling individuals aged 80 and above were taking anticholinergic drugs (22), while a German study focusing on elderly patients (aged 65–85 years) with multimorbidity found a prevalence of 54%, as determined by the GABS score (23).

Only a few studies investigated the aACh drug use among Memory Clinic outpatients, with reported prevalence rates ranging from 16 to 68% across different aACh scales and scores (16, 19, 24, 25). The prevalence found in our study is in line with existing literature, highlighting the frequent exposure of elderly patients to anticholinergic medications despite recommendations against their use in this population.

Multivariate regression analysis showed that in our patients, ACB score was independently associated with high NPI score, even after adjusting for factors such as age, sex, number of drugs, lost IADLs, and MMSE results. This finding is in agreement with prior studies performed in Memory Clinic patients (19, 24), and suggests that anticholinergic drug exposure is associated with BPSD. The reason of this association is unclear. The most obvious explanation could be that patients affected with BPSD are commonly treated with antipsychotic and antidepressant drugs, which have high ACB scores. However, the link between BPSD and aACh drugs seems to be more complex. Muscarinic neurotransmission is implicated, indeed, in behavioral control in humans. M1 and M4 are the most represented isoforms of muscarinic receptors in the brain being highly expressed in regions implicated in the pathogenesis of psychosis including frontal cortex, dorsal and ventral striatum and hippocampus (26). Intriguingly, muscarinic receptor distribution largely overlaps with dopaminergic pathways implicated in psychosis. As a matter of fact, M1 and M4 muscarinic receptors negatively modulate dopaminergic neurotransmission in these regions with the former operating a top-down (from cortical to subcortical areas) and the second a bottom-up inhibition (from subcortical to cortical areas) (26). Therefore, the stimulation of central muscarinic receptors is expected to exert antipsychotic effect and its inhibition to promote psychosis. As a matter of fact, a wealth of evidence supports the hypothesis of a dysfunction of muscarinic neurotransmission in schizophrenia (26, 27) and recently FDA approved Xanomeline Trospium, the first muscarinic agonist for the treatment of this disease (28). Intriguingly, the first demonstration that this drug could exert beneficial effects in human psychosis was obtained in 1997 in patients with AD and BPSD (29). These considerations suggest that a high ACB score could intrinsically worsen BPSD and reduce the response to antipsychotic and antidepressant drugs in this condition.

Considering the profound impact of BPSD on patients and caregivers, the deprescription of cholinergic antagonists should be considered in patients with behavioral disorders (30). Minimizing anticholinergic burden by opting for alternatives with similar therapeutic effects but devoid of anticholinergic properties may lead to a reduction in the frequency and severity of neuropsychiatric symptoms in patients with cognitive impairments. Specifically, a low ACB score should be included among the criteria for choosing among the various antidepressants and antipsychotics drugs available for the treatment of BPSD.

Our study population of memory clinic patients was on polypharmacy, with an average number of drugs of 6.4 per patient. This may contribute to BPSD through several complex drug interactions which may or may not involve the cholinergic system. As an example, benzodiazepines are known to cause confusion and worsen cognitive function in elderly patients by enhancing CNS depressant activity and GABAergic tone. Other drug classes such as diuretics may lead to significant electrolyte imbalances and contribute to the BPSD, depending on their dosages and duration of use.

**Figure 1:**
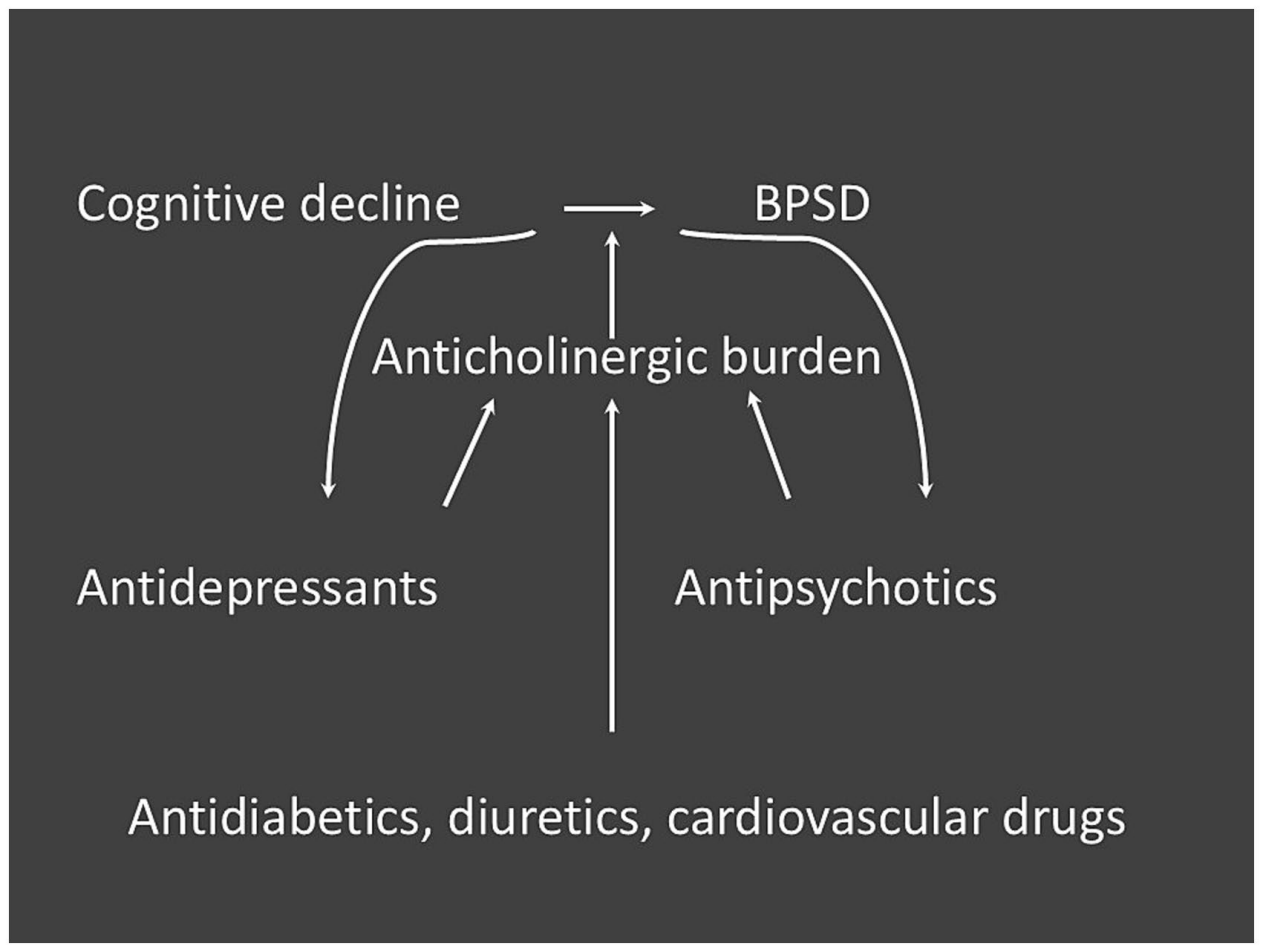
Possible relationship between drug classes with anticholinergic burden and BPSD in subjects with cognitive impairment.

Selecting medications with lower anticholinergic effects may mitigate associated side effects. However, it’s noteworthy that non-psychotropic drugs with anticholinergic properties, such as metformin and furosemide, are also frequently prescribed to elderly patients. This suggests that the geriatricians should carefully revise the therapy of their patients affected with dementia by choosing, when it is possible, drugs with no anticholinergic properties and replacing drugs with high ACB score with drugs with the lowest possible score (Figure 1) (19). Anticholinergic burden assessment tools can greatly help to achieve this goal.

Furthermore, our findings revealed a correlation between high anticholinergic exposure and functional as well as cognitive decline, consistent with prior literature (11, 16, 19, 24, 31). Avoiding anticholinergic drug usage could thus lead to several benefits for elderly people.

### 4.1 Limitations

Several methodological limitations need to be acknowledged when interpreting our study findings. The cross-sectional design precluded determining the directionality of the relationship between anticholinergic drug intake and the presence and severity of behavioral and psychological symptoms of dementia. However, existing evidence and recommendations suggest a plausible link between aACh drugs and BPSD. To explore this relationship further, longitudinal studies conducted on randomized population samples are warranted.

Also, it could be that patients with cognitive problems who attend a Memory Clinic were more likely to have BPSD, since these have a strong effect on both patient’s and caregiver’s quality of life, and therefore they are more inclined to seek medical assistance. Consequently, both the prevalence of BPSD and the potential influence of aACh drugs in precipitating symptoms may have been overestimated.

There are many methods for quantifying anticholinergic burden, which are heterogeneous, so that comparison between studies can be problematic and consequently it is difficult to draw conclusions in systematic reviews (11). Furthermore, anticholinergic scales, including the ACB score used in this study, do not take drug dosage or duration of therapy into account and may include medications with limited clinically relevant adverse cognitive effects. Other scales, such as the Belgian Muscarinic Acetylcholinergic Receptor ANTagonist Exposure Scale (MARANTE) take into account dosage information; however, they have information on a limited number of internationally available drugs (32). In the present study, we used the ACB score, which is based on the ACB (7) and GABS scales (10), considered in a recent review to be the scales with the highest quality studies in the literature; however, there is currently a need for comparison studies between the different anticholinergic scales in order to define the most suitable scale to be used for clinical practice and future clinical studies (8).

The observed results of the present study may be generalized to other populations presenting with similar characteristics such as outpatients of memory clinics with cognitive complaints, at all stages of diagnosis. However, our findings may not be applicable to other populations.

## 5 Conclusion

Our results suggest that aACh drugs might be a risk factor for the onset of BPSD in patients with dementia, independently from the severity of cognitive decline. aACh drugs should be avoided as much as possible in patients with dementia, preferably substituting them with alternatives with fewer aACh properties. Heightened awareness regarding both the aACh characteristics of commonly prescribed drugs among the elderly and their potential impact on cognition, behavior, and functional abilities in individuals with cognitive impairments is essential. Our findings suggest that not only psychotropic drugs (antipsychotics, antidepressants), but also drug classes such as antidiabetics might contribute to aACh burden in the elderly with cognitive impairment. By reducing anticholinergic burden, it is possible to mitigate the onset and severity of behavioral symptoms in dementia patients. There are currently several validated anticholinergic scales available, including country-specific ones (10, 33), which can be valuable tools for clinicians in this regard.

## Data Availability

The raw data supporting the conclusions of this article will be made available by the authors, without undue reservation.

## Footnotes

^1^ www.acbcalc.com
